# Mind the Gap: Understanding Differences Between Sexual and Reproductive Health-Related Legal Frameworks on Paper and in Practice

**DOI:** 10.3389/fgwh.2022.838976

**Published:** 2022-05-06

**Authors:** Laura Ferguson, William Jardell, Miles Lambert-Peck, Lillie Guo, Sophia Lopez, Violeta Canaves, Emilie Filmer-Wilson

**Affiliations:** ^1^Institute on Inequalities in Global Health, University of Southern California, Los Angeles, CA, United States; ^2^United Nations Population Fund, New York, NY, United States

**Keywords:** sexual health, reproductive health, legal framework, human rights, law and policy, SRHR

## Abstract

**Introduction:**

UNFPA recently developed a composite indicator to assess sexual and reproductive health (SRH)-related laws as part of the Sustainable Development Goals monitoring framework (Indicator 5.6.2). However, there is still little understanding of how best to ensure a supportive SRH-related legal framework can improve SRH outcomes. This research draws on country case studies (Colombia, Malawi, Uruguay, Zambia) to provide more generalizable lessons on the processes by which these laws are translated into practice and their impact on lived realities.

**Methods:**

Peer-reviewed and gray literature on laws and policies related to maternity care, contraception, sexuality education, HIV and HPV was reviewed. Key informant interviews were carried out with 8–16 people in each country, including representatives of government, civil society and academia to understand factors affecting implementation of relevant laws and policies. Findings were thematically analyzed by country and contextualized within each country's score on Indicator 5.6.2 and relevant SRH outcome data.

**Findings:**

Across these countries, some common organizational steps help move from laws on paper to impacting people's lives including budget allocation, development of technical guidance, health worker training, population awareness creation and demand generation. It is also important to address sociocultural challenges such as entrenched inequalities, conservative cultural and religious beliefs and the potential existence of customary law. Challenges can be encountered across all these steps and can vary based on the area of SRH: implementation of laws to reduce maternal mortality is generally less controversial than laws around abortion, often making the latter harder to implement. Local specificities in structures, systems and cultures bring opportunities and challenges, highlighting the need for tailored actions.

**Discussion:**

A legal framework supportive to SRH is critical, particularly in the face of backlash against sexual and reproductive rights, but alone it is insufficient. Understanding that a generic pathway exists for moving laws into practice is a critical starting point for exploring the specificities of each national context as a way of identifying entry points for action. These findings can be used to inform advocacy and monitoring to help ensure that the potential benefits of supportive SRH-related laws can be realized in these four countries and around the world.

## Introduction

In every country, sexual and reproductive health (SRH) is governed by a complicated array of laws developed over decades, which often emanate from different parts of the government. A supportive legal framework is essential for promoting access to SRH services, informed decision-making, and improved SRH outcomes. However, the true impact of the legal framework depends on its implementation, which requires sustained effort by a range of stakeholders within and outside the government. Analyzing relevant laws only on paper is insufficient to understand their impact on people's lives.

Even where a specific service is legally available, there may be restrictions in the law regarding who can access it, excluding, for example, minors, non-citizens, or women who have not secured spousal consent. Furthermore, conflicting laws can leave people confused as to what their legal entitlements are while service providers can be unsure what their legal liability might be for providing certain services. Plural legal systems may also exist (such as the co-existence of customary or Sharia law alongside statutory law) that provide conflicting frameworks for the provision and uptake of SRH services.

Understanding each country's hierarchy of laws is important for understanding how they all fit together from a legal perspective. Public perception of an SRH issue can be impacted by the types of law it is governed by, i.e., whether it is governed by a country's Health Code, exists as a separate law altogether, or is a provision in the country's Penal or Criminal Code, as is often the case with abortion (1–4). In the latter situation, abortion can be relegated to an illegal act and framed within the confines of criminality, making it more difficult for health workers to approach the topic from a public health or rights-based perspective.

The content of some SRH-related legislation can be open to interpretation, meaning that further processes may be required for clarification including government-issued directives as well as court rulings.

Ensuring that supportive laws can translate to improved health outcomes is contingent on political will. In some countries, implementing SRH-related laws are low-priority, stalling health improvements for their citizens, especially women and youth. In some cases, this is due to institutional factors that impede effective implementation, while in other cases sociocultural norms may hamper implementation. Irrespective of the reason, when governments and others show an unwillingness to implement SRH-related laws, deficiencies in legal awareness, service provision and service uptake may occur (5, 6). In contrast, when governments provide early, coordinated, and continued support for these laws, rapid diffusion of legal awareness and service availability is possible, which promotes improved SRH outcomes. Furthermore, collaboration with international and local NGOs, advocates and activists, and partners in the private sector provide for multi-sectoral implementation of the law (7).

Functional accountability mechanisms can help promote implementation, including the judicial system as well as more informal mechanisms such as civil society advocacy.

A range of measures and indicators exist that capture information on the existence and, to some extent, content of SRH-related laws. UNFPA recently developed a composite indicator to assess SRH-related laws as part of the Sustainable Development Goals monitoring framework (SDG Indicator 5.6.2) (8). Indicator 5.6.2 covers four dimensions of SRH: maternity care (maternity care, lifesaving commodities, abortion, and post abortion care), contraceptive services (family planning, consent and emergency contraceptives), sexuality education (topics and curriculum), and sexual health and wellbeing (HIV counseling, testing and treatment and HPV vaccines). The indicator is the first global indicator that captures information on the existence of supportive laws, any restrictions within laws (e.g., required parental consent for accessing services), and the existence of plural legal systems that might affect the impact of laws.^1^

But it is harder to capture information on the degree to which these laws are implemented, and therefore the level of impact they might have on people's health and lives. The existence of a supportive law does not necessarily translate into the provision of services, nor does it ensure that services are accessible for populations who need them most. Challenges with attribution impede quantitative analysis of the impacts of law on SRH outcomes but qualitative exploration can help understand different dimensions of legal implementation.

This research is part of a larger evaluation that included four country case studies—Colombia, Malawi, Uruguay and Zambia, each of which performed well on SDG Indicator 5.6.2, to understand the broader dynamic processes by which these laws are translated into practice and their impact on lived realities. For the purposes of this study, SRH is understood within the parameters of SDG Indicator 5.6.2. In this paper, challenges and facilitators to implementation across the four countries are thematically explored with a view to informing future actions to help maximize the contribution of supportive laws to positive SRH outcomes in multiple contexts. The aim is to use the key findings and lessons learned from the four countries to provide recommendations that can help inform governments and civil society across a wider range of countries to maximize the potential benefits on people's lives of a supportive SRH-related legal environment.

## Methods

Colombia, Malawi, Uruguay and Zambia were selected for a United Nations Population Fund (UNFPA)-commissioned study. The purpose was to explore the factors affecting the implementation of supportive SRH-related laws in low- and middle-income countries so it was important to choose countries that had scored well on at least some of the components of SDG Indicator 5.6.2, as well as a mix of countries that could provide comparisons within and across regions with different legal, epidemiological and cultural profiles.

### Desk Review

A literature review was carried out of peer-reviewed and gray literature. The peer-reviewed literature review was carried out in Scopus and covered English-language publications since 2000 relevant to this topic. We developed a comprehensive search strategy that included standardized key terms related to the four categories encompassed in SDG indicator 5.6.2: maternity care, contraception, sexuality education, HIV and HPV (Supplementary Material 1). All articles that met these search criteria went through a title review for relevance, an abstract review for context, and a full document review, with articles not meeting criteria being removed at each phase. Articles that met criteria were included in a data extraction matrix where data was systematically extracted on the relevant elements of SRH. This included information on the existence of supportive laws, the existence of legal barriers, restrictions within the law, societal, social and structural factors affecting legal implementation, impacts of existing laws, existence of plural legal systems, and conflicting laws within the broader legal environment. Successes in changing laws and lessons learned were also extracted where available.

The gray literature review included reports and publications authored by governments, civil society organizations, universities, and international organizations that explicitly addressed laws relating to maternity care, contraception, sexuality education and HIV and HPV services. Through the support of the local UNFPA offices and national government officials in the four case study countries, relevant documents for this analysis were identified. Information was extracted relating to: legal guarantees, legal barriers, implementation efforts, implementation barriers, and useful epidemiological information as relevant to components within SDG indicator 5.6.2.

In total, 262 articles were included in the final peer-reviewed literature review: 144 relating to maternity care, 80 relating to contraception and family planning, 12 relating to comprehensive sexuality education and 26 relating to sexual health and wellbeing (HIV and HPV). In addition, 41 gray-literature documents were reviewed, and relevant content extracted. The topic most covered across the literature was abortion, and least attention was given to HPV vaccination.

### Key Informant Interviews

Key informant interviews were carried out with a minimum of 8 and a maximum of 16 people in each country. A purposive sample of key informants was identified by the UNFPA country offices based on who would be well situated to provide relevant information around laws and policies regarding SDG Indicator 5.6.2. Interview participants made recommendations for additional participants, who were also included. Key informants included medical and public health professionals, lawyers, Parliamentarians and advocates. Everyone was interviewed in their professional capacity; patients and adolescents were not interviewed.

Across all four study countries, 43 people were interviewed, as detailed in Table 1.

**Table 1:** Interview participants.

**Country**	**Government**	**Professional associations**	**Civil society**	**Academia**	**UN organizations**	**Total**
Colombia	7	1	5	0	3	16
Malawi	3	0	5	0	0	8
Uruguay	5	1	3	2	0	11
Zambia	2	0	4	0	2	8
Total	17	2	17	2	5	43

The same semi-structured interview guide was used in all countries, translated into Spanish for the interviews in Colombia and Uruguay. The guide was developed based on preliminary findings from the desk review and tailored to each country context. The interview guide (Supplementary Material 2) was used to generate discussion on the general context within each country, key actors involved in implementation of SRH-related laws, recognized barriers and facilitators for implementation and suggested actions for improving implementation of relevant laws. Questions were tailored to an informant's area of focus as well as the roles that their organization plays.

Interviews were carried out over Zoom between March and May 2021 by bilingual qualitative researchers with experience working on SRH in both regions. Detailed notes were taken during the interviews, usually by a research assistant. Researchers determined that saturation had been reached when interviews stopped yielding substantial new information on themes of interest and when there were no new suggestions for people to interview. Coding was not used for interview analysis. Instead, all interviews were thematically analyzed by country, using themes derived from the initial literature review as well as additional information that emerged in the interview data. Comparative analysis was carried out within regions and then across all four countries.

Findings are contextualized within each country's score on SDG Indicator 5.6.2. Data for this indicator were collected as part of the UN's 12th Inquiry on Population and Development and analyzed by UNFPA. The indicator is a percentage scale up to 100, indicating a country's status regarding the existence of national laws and regulations to guarantee full and equal sexual and reproductive health and rights (SRHR). Negative values reflect the existence of more legal restrictions than supportive laws. This may mean, for example, that a country has restrictions on abortion, such as requiring a husband's consent for a married woman to access abortion, and that it criminalizes obtaining an abortion (8).

## Results

Table 2 provides an overview of SRH-related laws in the four study countries, as reported for SDG Indicator 5.6.2.

**Table 2:** Overview of SRH-related laws (8).

**5.6.2 Components/indicator 5.6.2—score (out of 100)**	**Colombia**	**Uruguay**	**Malawi**	**Zambia**
*Section 1: maternity care*	*92*	*96*	*38*	*88*
Maternity care	100	100	25	100
Life saving commodities	92	85	100	100
Abortion	75	100	−50*	50
Post-abortion care	100	100	75	100
*Section 2: contraceptive services*	*100*	*100*	*93*	*78*
Contraceptive services	100	100	80	60
Contraceptive consent	100	100	100	100
Emergency contraception	100	100	100	75
*Section 3: SEXUALITY EDUCATION*	*100*	*100*	*100*	*100*
Sexuality education curriculum laws	100	100	100	100
Sexuality education curriculum topics	100	100	100	100
*Section 4: HIV and HPV*	*100*	*100*	*90*	*100*
HIV: counseling and test services	100	100	80	100
HIV: HIV treatment and care services	100	100	80	100
HIV confidentiality	100	100	100	100
HPV vaccine	100	100	100	100
**Overall 5.6.2 score**	**97**	**99**	**76**	**91**

All of these countries scored well on the indicator, with all of them scoring full marks for their Comprehensive Sexuality Education (CSE)-related legal environment, and the lowest scores seen in the area of maternity care. Yet, these high scores do not necessarily translate into good SRH outcome indicators (Table 3).

**Table 3:** Indicator 5.6.2 scores and select SRH outcomes for each country.

**Country**	**Colombia**	**Uruguay**	**Malawi**	**Zambia**
**5.6.2 Section 1—maternity care**	**92**	**96**	**38**	**88**
MMR (maternal mortality ratio)	83	17	349	213
**5.6.2 Section 2—contraceptive services**	**100**	**100**	**93**	**100**
Contraceptive prevalence rate, modern—(women 15–49)	60	55	47	35
% demand satisfied w/modern contraception (women 15–49)	87	87	77	67
**5.6.2 Section 3—sexuality education**	**100**	**100**	**100**	**100**
Adolescent fertility rate	61	36	138	135
% of women married by age 18	23	25	42	29
% female adolescents w/ comprehensive, correct knowledge of HIV (age 15–19)	27.7	73.5	38.9	40.5
**5.6.2 Section 4—HIV/HPV**	**100**	**100**	**90**	**100**
% of women LHIV who know status	53		94	93
% of men LHIV who know status	64		86	87
% of women LHIV who are virally suppressed		90	94	91
% of men LHIV who are virally suppressed		75	93	90
HPV vaccine complete coverage—women	39	38		

Across most of these SRH outcome indicators, the differences between regions appear greater than the differences by score on the relevant SDG indicator section. Traditional norms around adolescent sexuality and child marriage differ between the two regions represented, as does the severity of the HIV epidemic, all of which might help explain some variability. However, the legal indicator scores are also useful. It is noteworthy that Uruguay, the country with the highest score on the maternity care section of Indicator 5.6.2 and the only country where prosecution of women for procuring an abortion is not allowed, has the lowest maternal mortality ratio. Malawi, which had a negative score on the abortion component of Indicator 5.6.2 has by far the highest maternal mortality ratio. This is not to say that abortion law is the sole determinant of maternal mortality, but that it might play an important role given what is known about higher maternal mortality in situations of unsafe abortion. This table highlights the fact that the mere existence of supportive laws, while critical, is not enough to guarantee good SRH outcomes. Thus, deeper exploration of the factors affecting implementation of these laws is warranted.

An interview participant in Colombia remarked:

“*Colombia has a very strong legal framework around sexual and reproductive rights and sexuality education… But the big challenge that we face is how to bring these laws to life. We don't need any more laws. We have very good laws but we have to ground them, to make them real in schools and in health facilities… We have to be able to reach everyone to ensure that they have the skill, knowledge and appropriate attitudes to guarantee everything the laws say so nicely.”* (KII C13)

Looking across these four countries, a wide range of factors affects the degree to which different SRH-related laws have been implemented, and variation is seen in implementation within each country. Yet some common factors affecting implementation of supportive laws emerged that can help inform SRH-related work more broadly. Across publications and interviews, some of the factors determining how much progress had been made with implementation include: when policies and guidelines were put in place, resources within the system to implement them, which other sectors are involved in the response to act on the social determinants of health, and on sociocultural factors such as traditional beliefs, customary law, local constructions of gender, and entrenched religious beliefs. It is useful to understand that a generic pathway to legal implementation exists, even as it must be tailored to each context.

Different factors requiring consideration along the pathway to implementation of relevant laws are explored below, starting with the organizational factors that influence duty-bearers' implementation of a new law, followed by an analysis of sociocultural factors that also shape these actions as well as those of communities and individuals. Lessons are drawn from across the four study countries, with individual examples used to illustrate a trend or an outlier as useful.

### Organizational Factors

#### The Broader Legal Environment

SDG Indicator 5.6.2 captures some useful nuance in laws such as specific situations in which abortion is decriminalized or if third-party authorization is required to access services, but additional details are also important that are not captured by this indicator. For example, the steps required by law to access abortion even in the situations where it is decriminalized can create substantial barriers. These can include mandatory reflection periods as well as the need for multiple consultations with a multidisciplinary team of health specialists (who may be very hard to find in rural areas).

Even where countries report the existence of supportive SRH-related laws under SDG Indicator 5.6.2, it is useful to look at a much broader range of laws that can also impact SRH outcomes. This might include, for example, laws that allow child marriage, that criminalize HIV exposure or transmission, and that criminalize certain behaviors including sex work, sex between men, and drug use (9).

Sometimes other laws might conflict with the laws reported under SDG Indicator 5.6.2. For example, the penal codes of both Colombia and Uruguay criminalize abortion even as the content of the penal code is now superseded in Colombia by the more recent Constitutional Court ruling and in Uruguay by the more recent Law on Voluntary Termination of Pregnancy, both of which decriminalized abortion in specific situations (10–13). In Malawi, discrepancies are also found between the Penal Code and the more recent Gender Equality act (14). This can be confusing for women and health workers alike and can lead to the avoidance of services to which women are legally entitled for fear of prosecution.

In Malawi, a Ministry of Education, Science and Technology policy forbids delivery of SRH services within 100 meters of a school, which negatively impacts access to contraception and teen pregnancy, and limits the effectiveness of comprehensive sexuality education as teachers cannot link students to nearby health facilities.

The broad range of laws that must be understood by health workers, educators and the general public to understand their SRHR and legal entitlements to services can create confusion and lack of confidence regarding the legality of providing or seeking certain services.

#### Policies and Technical Guidance

Policies and technical guidance documents are often used to add operational guidance to laws, helping to ensure that those responsible understand their responsibilities under current laws. In Malawi and Zambia, where the SRH-related legal framework is far less comprehensive than in Colombia and Uruguay, policies and strategies are particularly important for helping duty bearers, especially health workers, understand their legal responsibilities in service provision.

Although Malawi's SRH policy itself is relatively progressive, the extent to which policy provisions can be used to safeguard women's rights in SRH is limited because there is no legal foundation for some of what is found in policies. The same is true for HIV in Uruguay because there is no HIV-specific law.

In 2006, Colombia enacted a decree, which adopted WHO guidance for health workers on safe abortion to guide the provision of quality abortion services (6). In 2013, the Court annulled this decree, leaving a void in technical guidance: health facilities are still required to provide abortion services but there are now no official guidelines on recommended methods of care (15).

#### Financing

Another critical step in legal implementation is the allocation (and expenditure) of a budget. This might be used, for example, to train duty bearers to fulfill new functions, raise awareness of new laws, disseminate information around new rights and responsibilities and purchase newly approved supplies or equipment. In all four countries studied, there is, however, no budget allocation automatically made when laws are passed so financial allocations rest on political priorities in annual budget processes, whether at national level, sub-national level or both. A civil society representative in Malawi noted that:

“*Once a law is passed, there's no budget. Usually it takes CSOs [civil society organizations] to push; if you're quiet sometimes it can sit for years without implementation.”* (KII M4)

There are examples where civil society advocacy has contributed to increased budget allocations such as in Malawi where the White Ribbon Alliance lobbied the Parliamentary Committee on Health to increase family planning budgets, leading to a 6% increase in the 2020/2021 Family Planning National Budget (16). However, in Zambia, a 2020 family planning scorecard found that the government allocation for family planning programs was only 1.4% of total need (17). Across Colombia, Malawi and Zambia, civil society reported challenges in tracking government budgets and expenditure for SRH with no standard method of accountability within the governments. Costs of SRH services and commodities can be a barrier to uptake, so governmental budget allocation is critical to promoting equitable access.

Looking beyond SRH services, it is important to understand health financing priorities in different countries. For example, in Malawi, health funding is skewed to secondary- and tertiary-level health services, with community and primary health care relatively under-funded. In Colombia, health insurance companies play a critical gatekeeping role for access to services: profit margins vary by intervention, incentivizing insurance entities to promote high-cost, high-return services rather than low-cost services such as family planning. At an even more granular level, the capitated payments do not account for the differing costs of, for example, different methods of family planning so health facilities prioritize those that are cheapest, limiting the range of methods available to women and often excluding long-acting reversible methods. Furthermore, insurance companies may orally communicate to clients requirements for service access that do not exist by law, such as required third-party authorization, to disincentivize the uptake of certain services.

#### Dissemination of Information on Laws and Rights

Across all four study countries, there is widespread acknowledgment that dissemination of information about SRH-related laws and rights is inadequate, particularly given that this has to be an ongoing activity as frameworks evolve, and it has to cover the whole country. As a result, people often do not know their rights, what services are available to them, or how to access them. This is particularly true among rural women who are very dispersed and may rarely gather in groups: many of them leave school very early, marry young and are not exposed to information beyond their immediate community.

In Malawi, the media, who have received substantial training around SRH, play an important role in dissemination of information and garnering of support around SRH and related laws, including in controversial areas such as abortion. However, in some places, governments' commitment to disseminating information on SRH-related laws may vary by topic. An interview participant in Colombia noted that the government has carried out many more media campaigns to support dissemination of maternal health laws than the right to abortion, which she noted they had not done at all. Similarly, participants in Uruguay described the biggest challenges with SRH-related information dissemination as lying in areas that remain socially stigmatized such as abortion, HPV and HIV. Civil society organizations play a key role in disseminating information on SRH-related laws across the study countries.

#### Health System Structure and Capacity

To differing degrees, all countries experience challenges with health system coverage and quality, particularly in hard-to-reach areas. Lack of basic services such as internet or electricity, fragmentation of services, and inconsistent commodity supplies are common challenges to SRH service provision across all study countries. A 2016 study determined that only 30% of facilities in Zambia could potentially offer basic abortion services, and far fewer could potentially offer comprehensive abortion services (3.7%), basic post-abortion care (2.6%) and comprehensive post-abortion care (0.3%) (18).

In addition, countries face both a shortage and a maldistribution of health workers, with rural and hard-to-reach areas most negatively impacted. In Malawi, 50% of doctors and nurses are stationed in the four central hospitals while the remaining doctors are stationed among 24 district hospitals with no doctors in any of the 328 health centers (19). In Colombia, strict controls exist limiting the number of university places for specialist medical training, exacerbating shortages of expertise across the country. In general, interview respondents described very low knowledge among health workers about women's rights and SRHR within the context of the law (20). Frontline health workers often perceive the law as very distant from their work, highlighting the inaccessibility of the language of the law. In-service training, in a context of limited resources, focuses on technical skills to address areas of high mortality. Addressing morbidity, laws or rights is rarely prioritized. Across all study countries, NGOs have supported the government to provide some in-service training on SRH-related laws but this has not been done systematically. In Uruguay, the doctors' union has also carried out relevant in-service training on these issues, which stands in contrast from the other three countries where relevant professional associations have not engaged in this work.

Where ambiguities exist in the law or health workers are unsure about the details of a law, they may make their own decisions about whether or not to provide services. This was most often seen in the areas of abortion and adolescents' access to services. For example, in the countries that allow abortions on the basis of preserving the woman's health, there may be very different interpretations of “health”—notably, whether or not mental health is included alongside physical health (21). Sometimes interpretation is done through the justice system while in other instances, it is institutions or individual service providers who do this, which can lead to large differences in which services are delivered and to whom. In addition, it has been found that some staff may hold biases against providing family planning for youth, based on beliefs including that contraceptives promote sexual activity and that youth should not access contraceptives until they have had one child (22).

Where a large proportion of health facilities are owned by religious bodies, as is the case in all four study countries, facilities may choose to not provide certain services even if they are guaranteed by law, thus severely restricting access. Individual providers may also refuse to provide certain services based on their religious convictions (see Box 1).

Box 1 **Conscientious objection.**

Conscientious objection to the provision of abortion services is widespread, particularly in Colombia and Uruguay, although increasingly also in Zambia. Although the law allows it in specific circumstances that vary slightly by country, the extent of conscientious objection goes far beyond what is allowed within national laws, with substantial impact on women's access to abortion services, particularly in rural areas.

#### Education System Capacity

Psre- and in-service training are critical for ensuring that educators are comfortable delivering the CSE curriculum but, across all four countries, both were deemed insufficient. Appropriate training materials are often not provided either. Lack of accountability allows teachers to pick and choose what they teach if they teach these topics at all (22–24).

#### The Judiciary

In Colombia, the courts have played a critical role in revising and interpreting SRH-related laws, particularly in the area of abortion, which was not the case in the other countries studied (25). It seems that, for this to be a useful avenue for advancing legal protections relating to SRH, different elements need to be in place: an independent judiciary with up-do-date knowledge on relevant laws, international human rights standards and public health; a cohesive civil society to bring cases to court; and mechanisms for accountability to ensure court decisions are fully enacted.

#### Decentralization: A Double-Edged Sword

In large and diverse countries, health systems are often decentralized. Even as capacity is sometimes an issue—in terms of both human and financial resources—, the autonomy afforded to local levels provides latitude to tailor implementation to local circumstances. However, as is often the case, there can be challenges with things “falling between the cracks” where local actors lack capacity and national authorities do not have the mandate to intervene. Each level of decentralization provides a decision point at which implementation of SRH-related laws might be ignored or prioritized. The system relies on “trickle down governance”, which has been hard to monitor down to the ground.

#### Inter-sectoral Collaboration

The topics covered under SDG Indicator 5.6.2 require collaboration not only between the health and education sectors, but also potentially Ministries of Youth, Women, Justice and others. In places where collaborations are strong and respective roles clearly delineated, there is evidence of successful interventions, including on CSE and HPV vaccination within schools for example (26, 27). Where collaboration is weaker, shortcomings in data availability, communication and coordination have negatively affected some interventions (28).

#### Monitoring and Evaluation

Monitoring and evaluation are critical to success—we have to measure the right things, regularly look at the data and use them to inform programming decisions. Across the study countries, priority-setting was often target-driven and only maternal mortality and morbidity and adolescent pregnancy were considered priorities for SRH. Without better indicators around maternity and post-abortion care, services may be mortality centric without consideration for the intrinsic quality of life dimensions necessary for holistic care. In addition, clearly disaggregated data are missing for many SRH services, which impedes the design and delivery of evidence-based policy and services. This informs not only budget allocation but also how much attention is given to operationalizing laws in different areas including prioritization of regulations, policies, and capacity building efforts. Inevitably, this results in the neglect of certain issues or populations. Many people recognize the impossibility of achieving, for example, targets around adolescent pregnancy without funding CSE, but data gaps impede evidence-informed action across all areas.

Civil society engagement in monitoring and evaluation is critical for accountability; where strong capacity exists for this, as is the case in Colombia and Uruguay in particular, CSOs can help influence what data are collected and how they are used. A civil society participant from Zambia highlighted the importance of a strong legal framework as a foundation for accountability:

“*Law is very important because it provides the parameters of our work, guides us and protects us – without the law we can't put on these pressures [to hold the government to account]. There are a lot of other players who could exploit a weaker legal framework.”* (KII Z1)

Under-funding of National Human Rights Institutions in some places impedes their ability to contribute to monitoring efforts.

#### Accountability

Across the study countries, CSOs play a watchdog role and work to try to hold the government to account. This has been observed through questioning the health budget and calling out the insufficient support for certain areas of SRH. As an example of social accountability by CSOs, many have produced scorecards that highlight areas of SRH where programs should exist. In Uruguay, respondents noted that civil society monitoring of policy has been one of the most useful, efficient, and effective national tools for promoting SRH.

However, this work can be challenging where mechanisms for accountability are fragmented or inefficient, and the strength and collective experience of civil society on SRH and legal matters is, again, seen as fundamental to the success of these processes.

All of the study countries also engage with international accountability mechanisms such as the Universal Periodic Review and United Nations Treaty Monitoring Bodies. Through these mechanisms, recommendations are regularly provided regarding strengthening of national SRH-related legal frameworks and their implementation (14, 29, 30). These can be used to bolster local advocacy efforts to hold governments accountable.

#### Political Will

An interview participant in Colombia noted that “*Everything depends on political will and what the government in power believes*” (KII 1), an opinion also shared by participants in other countries. If the government does not agree with SRH-related guarantees in law, they can stop services and programs or create such bureaucracy that even if programs exist on paper nothing can ever be achieved. With elections every 4–5 years, government changes are frequent and there can be large ideological differences between national governments, and local governance structures, which affect the degree to which SRH-related laws might be implemented. This is particularly true with regard to “controversial” issues as it is seen as a political risk for governments to support them. A governmental official in Malawi noted:

“*In the political sector people are worried about re-election as abortion can be seen as anti-culture or religion. Back in the village, traditional leaders are against it in some quarters so MPs [Members of Parliament] are not ready to support even if they want to as individuals. Political parties want the votes and anything that is questionable in the eyes of the people, they don't want to support that.”* (KII M3)

Prevailing political winds shape not only governmental action but also the degree to which civil society might want to push different topics, taking advantage of opportunities where they exist but also treading carefully in times where retrogression seems possible.

### Sociocultural Factors

The ways in which sociocultural factors affect implementation of SRH-related laws are context-specific, with varying influence of these factors across and within countries. This section focuses on drawing out major trends in how sociocultural factors affect implementation of SRH-related laws across the four study countries, recognizing that there is, of course, important specificity within each country that is important to understand.

#### Inequalities

There are evident inequalities in health outcomes as well as in the implementation of SRH-related laws across different populations and parts of each country. Not only does this create a challenge for the interpretation of national-level statistics, it also complicates efforts to understand the impact of laws on health outcomes as this appears to vary widely within countries. Understanding these inequalities is critical to identifying potential gaps in legal implementation that might be minimized with directed investment.

Across all countries, people of low socio-economic status and those living in rural areas were considered among the hardest to reach in terms of ensuring the benefits of legal protections. Social constructs of gender are also a critical barrier for women: the four case study countries comprise patriarchal cultures, creating challenges for women with regard to autonomous decision-making, access to resources and even access to information. Taboos surrounding adolescent sexuality, particularly in Malawi and Zambia, continue to impact how adolescents might be treated in health facilities and, as a result, their willingness to seek services. A similar lack of support is also found in the community, often leaving adolescents with few trusted sources of SRH-related information let alone services.

#### Religion

Across the four study countries, religion plays an important role in the support given to the implementation of SRH-related laws and services. Even where there is a clear separation of church and state on paper, this often is not reflected when laws move from paper to implementation. The Catholic and Evangelical churches have traditionally been opposed to addressing some areas of SRH, affecting policies and even institutions such as health facilities and schools. As stated by an interview participant in Zambia, people with strong religious beliefs may hold top government positions, including in Parliaments, and participate in key decision-making spaces, into which they bring these beliefs:

“*Cultural norms infiltrate policies because policy-makers come from the community. Sexuality is taboo. Rarely do people, policy-makers, parents, communicate around this – we started a bit too late to open the dialogue around CSE and SRH, which has led to excess HIV.”* (KII Z1)

In Colombia, at least 10 of the major universities have religious origins, so when it comes to, for example, abortion, contraception, sexuality, gender and sexual violence, they teach in accordance with religious beliefs or they do not tackle these topics at all.

Even as, particularly in small communities, speaking out about controversial issues such as abortion and family planning can lead to stigma, discrimination and isolation, female leaders are emerging who want to learn about SRH-related laws and rights. With appropriate support, these women might help catalyze change in their own communities. Where churches are receptive to addressing SRH within their communities, this can greatly facilitate community acceptance of such programs. Some points of commonality have been identified through committed dialogue, and people on both sides continue to find ways to collaborate even on controversial issues. Sensitizing religious and traditional leaders continues to be considered a central tenet of all SRH efforts, including the need to situate discussions with the existing traditions of a given community and illustrate the importance of addressing SRH broadly. In Uruguay, participants noted the importance of the strong legal foundation for SRH in these discussions as it provides an umbrella for all action, protecting health workers, teachers and institutions so that they can work on SRH with less chance of retribution from conservative actors.

#### Cultural and Gender Norms

Patriarchal norms that limit women's autonomy and independent actions were universally described as a factor influencing the content of SRH-related laws and policies as well as a strong barrier to women's uptake of SRH services where these are available.

“*Even as we disseminate this [SRH] information, we're fighting against the culture. Our patriarchal system is still very strong so for some women they feel their husband must allow them to go and access family planning… must give them the permission. They don't know they have the right to self-protect against STIs. The issue of submission is very strong. We let them know they have this right.”* (KII Z2)

The value placed on motherhood within many cultures creates stigma around family planning and abortion. An academic in Uruguay described this:

“*Abortion stigma is still a very important deterrent. There's a complex set of factors influencing how decriminalization is understood within communities. In some, predominantly low SES [socioeconomic status] and highly religious, communities, the discourse of motherhood as a woman's destiny persists and motherhood is culturally very highly valued. These are the areas with the highest adolescent birth rates, most social exclusion, most neo-Pentecostal churches, religious social programs and Catholic churches.”* (KII U9)

#### Cultural Diversity

Cultural and linguistic diversity can create challenges with regard to understanding sexuality across the countries, with some interview participants voicing concerns about how SRH-related questions are asked in different languages. This can impact not only the understanding of SRH-related priorities but also the acceptability of services.

Where a dual-legal system exists, customary law (which is administered by male traditional leaders) may create limitations around enforcement of statutory laws. Although SDG Indicator 5.6.2 does not include child marriage laws, this is an area where customary law can diverge from the formal legal system, with substantial negative impacts on implementation of the formal law and on adolescent education and SRHR, particularly among females. For example, in Zambia, although the official age of marriage is 18 as per the Constitution, traditional statutes, which vary across the country, allow girls to be married off by their parents when they begin to menstruate or at a set age below 18 (31). Interview participants reported that child marriage is still very prevalent, particularly in certain regions such as the southern province. A similar situation was described in Malawi:

“*People don't believe in family planning in the community. Chiefs believe the more people they have the more power they have. Parents are encouraging girls to marry early. Teenage pregnancy is seen as natural.”* (KII M6)

Further, participants noted that in many rural areas, people are not aware of the statutory laws that exist. This can create tension between health workers and others who offer services who may face legal barriers in providing services. In Malawi, by-laws were created at community level with the aim of improving maternal health, but some contradict national laws or do not align with national-level health policies and objectives, and they may have the unintended consequence of exacerbating health inequities (32).

Even where there is no official plural legal system, cultural norms around access to services can still influence certain populations' access to SRH services. For example, in some indigenous communities in Colombia, the use of western health services is unacceptable. Indigenous women who use an indigenous health facility and want to access abortion services have to seek permission from a male-dominated council of indigenous leaders where it is often denied. So, although these women should have access to free public services, they often have to go to other cities and access abortion through CSOs or the private sector.

## Discussion

Looking across these four study countries, there is a clear set of activities that is required to ensure that SRH-related laws can be implemented and maximize their positive impact. However, in the context of differing political, legal and health systems as well as diverse sociocultural settings, how these activities are implemented, which ones are prioritized and who is best placed to implement them vary greatly.

SDG Indicator 5.6.2 gives valuable insight into SRH-related laws and can be usefully situated within the broader legal framework. Then, a deeper exploration of factors affecting legal implementation can identify areas for action to maximize the benefits of a supportive SRH-related legal environment. SRHR are governed by a range of laws, which originate from different parts and levels of the government. Understanding each country's hierarchy of laws is important for understanding how they all fit together from a legal perspective, especially if some laws contradict others. Devolved systems where sub-national laws and regulations may also exist create an additional layer for analysis.

Within each country, a structured pathway to implementation could usefully be required for each new law influencing access to or provision of SRH services. This might include details around budget allocation, policies and technical guidance that will be needed, training of health workers, health system strengthening to ensure consistent availability of supplies, supportive supervision, awareness-raising within communities about the implications of the new law and a plan for monitoring and accountability. While the details of this might vary by law, each law could still require that a work plan be set out within a specified time period of the law being passed that considered each of these steps. This can facilitate future evaluation of implementation, identify bottlenecks and provide multiple entry points for action. A budget might also be automatically allocated to ensure that implementation activities can be carried out.

The low levels of legal awareness—both on the side of the provider and on the side of the patient—found across the four study countries has also been shown in other studies to be a barrier to accessing safe, quality services (3, 33–35). This is especially true amongst young, unmarried, rural-dwelling, low-income women with little education, for whom legal awareness tends to be lower than their older, married, highly educated, urban-dwelling, and higher-income counterparts (36–40). Low awareness of the law amongst women and providers encourages hesitancy among both groups–reluctance to seek services for the former and reluctance to provide services for the latter, both borne of fear of legal repercussions.

Previous studies have examined how shortcomings within the health system can impede access to SRH services, meaning that SRH-related laws are not being fully implemented. This includes attention to the inaccessibility of services due to long distances to health facilities and lack of supplies and equipment. Inadequate numbers of appropriately trained health workers, particularly in rural areas, have been found to impede the provision of certain SRH services, particularly abortion services (5, 41, 42). Several countries have introduced task-shifting, a process allowing mid-level healthcare workers to complete certain clinical tasks including some relevant to SRH and HIV, effectively removing some responsibilities from physicians (33, 36, 42).

Other research has also explored the potential conflict between personal religious or cultural beliefs and the provision of specific SRH services such as family planning for adolescents, CSE or abortion services (2, 33, 43, 44). Many countries report high proportions of providers who conscientiously object to providing a variety of SRH services, and this percentage has increased in some places (3, 45, 46). The right to conscientiously object is commonly detailed in national legislation, almost always accompanied by a requirement to refer the client to another provider. However, some health workers consider referral antithetical to their religious or moral beliefs. Moreover, the referral process can be time-consuming and cumbersome, and may ultimately obstruct access as most people seeking abortions are legally bound by time constraints.

The ability to legally object is becoming more widespread amongst healthcare professionals—for example, there has been an increased visibility of pharmacists who conscientiously object to providing contraception, including emergency contraception, medication abortion, or other drugs (47). Additionally, the types of services for which a provider can conscientiously object may be increasing; post-abortion care, for example, is more commonly being accepted into the array of services for which a provider may conscientiously object (48). Oftentimes, it may be unclear who can conscientiously object, although it has been documented that healthcare providers including anesthesiologists, nurses, and patient transportation staff have done so (5). In some cases, entire facilities may de facto invoke conscientious objection if administrators or a large proportion of providers refuse to dispense abortion services, as in Colombia and Uruguay, even when this is not legally provided for (39).

There is relatively little attention in previous public health literature to the roles of healthcare professional associations (or unions) and the judiciary in implementation of SRH-related laws. We found the former to be useful for helping build the capacity of health workers to deliver services according to emerging laws in Uruguay, and the latter to be invaluable for shaping the abortion-related legal framework in Colombia. Relatively little has also been written about the role of universities in training health workers on their legal responsibilities.

Not only does the legal framework itself matter but systemic factors such as how the health system is financed, what role the private sector plays the extent to which services are within reach of all populations and the ways in which inter-sectoral collaboration are fostered are all critical to understand in order to determine how best supportive laws should be implemented.

Beyond these systemic factors, sociocultural factors are a well-recognized challenge to the implementation of some SRH-related laws. Just as this study found socioeconomic inequalities to impact women's ability to benefit from the legal protections available to them, other studies have found that some women and girls may not have access to SRH education if they did not have the opportunity to attend secondary school (37). In societies where women are further disenfranchised by their marital status, unmarried women have even less access to family planning and contraception (7, 49). In communities where, irrespective of the law, family planning decisions are primarily made by mothers-in-law and husbands, women may have little say in important and invasive matters regarding their bodies, such as the number of children planned, sterilization, and contraceptive use (50, 51).

This study found religious beliefs continuing to pervade conversations about SRH services, especially abortion. In many settings, religious norms remain relevant and prominent even when new SRHR-related laws are passed, and religious leaders often play an important role in the acceptability of these laws to communities. For example, although widely influential religious groups in Ethiopia did not oppose the country's 2005 abortion liberalization on a federal or legal level, they continued their anti-abortion advocacy through community-based channels, such as through priests or sheiks. Conversely, the secular nature of many states has been used as an argument in favor of the provision of SRH services. In Tunisia, where 99% of the population is Muslim, the government opted for a secular approach and first liberalized its abortion laws in 1965 (52). Similarly, established separation of church and state can allow abortion advocates to hinge their arguments on the secular nature of the state, as seen in Mexico (53).

Persistent taboos about SRH, particularly among adolescents, continue to impede the implementation of some supportive SRH-related laws. The challenges of culture change are well-acknowledged but there are examples of success that can be built on to help open up dialogue, which will be critical to shaping societal attitudes moving forward.

Although the generic pathway to legal implementation is a useful starting point, the major differences in challenges at different points of this pathway across these four country case studies highlight the need for a detailed understanding of local context to inform how best to take advantage of supportive legal frameworks, where they exist, to create positive changes in people's SRH outcomes. Beyond the structure of the health and legal systems and the range of factors explored here, understanding the roles and capacities of key stakeholders will also be key. With this many factors affecting the potential implementation of SRH-related laws and their impacts on SRH outcomes, it is unsurprising that there is no clear association between the existence of a law and good outcomes. A set of measures could usefully be developed capturing different steps along the pathway to implementation to help understand, in different contexts, where to focus efforts to improve implementation and to document successful approaches to this work.

## Conclusion

This study substantiates findings of previous studies across a range of settings and systematically analyzes how, across four different contexts, information on factors affecting legal implementation can be used to inform policy and programmatic action. Its conclusions point to useful steps that all countries can consider to maximize the positive impact of a supportive SRH-related legal framework. They also highlight some of the major factors to be assessed to identify who might be best placed to take on different elements of implementation such as the strength of the health system, the judiciary and civil society. SDG Indicator 5.6.2 provides a useful starting point for using the law to improve SRH outcomes; applying these additional analyses to inform action can help countries meet multiple international commitments, including their SRH-related obligations under the SDGs.

## Data Availability Statement

The raw data supporting the conclusions of this article will be made available by the authors, without undue reservation.

## Ethics Statement

Ethical review and approval was not required for the study on human participants in accordance with the local legislation and institutional requirements. Written informed consent for participation was not required for this study in accordance with the national legislation and the institutional requirements.

## Author Contributions

LF and EF-W conceptualized this study. LF led data collection and analysis as well as the drafting of the manuscript. WJ participated in data collection and analysis and drafting of the manuscript. ML-P and VC participated in data collection. SL and LG participated in data analysis. All authors have read and approved the final manuscript.

## Funding

This project was funded with generous support from the United Nations Population Fund.

## Conflict of Interest

The authors declare that the research was conducted in the absence of any commercial or financial relationships that could be construed as a potential conflictof interest.

## Publisher's Note

All claims expressed in this article are solely those of the authors and do not necessarily represent those of their affiliated organizations, or those of the publisher, the editors and the reviewers. Any product that may be evaluated in this article, or claim that may be made by its manufacturer, is not guaranteed or endorsed by the publisher.

## Footnotes

^1^The full questionnaire is available at: https://www.unfpa.org/resources/sdg-indicator-562-questions.
