# Supplementary material for: Mind the Gap: Understanding Differences Between Sexual and Reproductive Health-Related Legal Frameworks on Paper and in Practice
Source: Front Glob Womens Health. 2022 May 6;3:838976. doi: 10.3389/fgwh.2022.838976 (PMC9120771; doi:10.3389/fgwh.2022.838976)
Supplement: Supplementary file 1 [file Data_Sheet_1.pdf]

Supplemental File 1 - Literature review search strategy:

TITLE-ABS ("Maternity Care") TITLE (law OR legal) AND PUBYEAR > 2000
TITLE-ABS ("Essential Medicines") TITLE (law OR legal) AND PUBYEAR > 2000
TITLE-ABS ("Abortion") TITLE (law OR legal) AND PUBYEAR > 2010
TITLE-ABS ("Legal status of abortion") AND TITLE (law OR legal) AND PUBYEAR > 2000
TITLE-ABS ("Post-abortion care") AND TITLE (law OR legal) AND PUBYEAR > 2000
TITLE-ABS ("Contraception") AND TITLE (law OR legal) AND PUBYEAR > 2000
TITLE-ABS ("Family planning") AND TITLE (law OR legal) AND PUBYEAR > 2000
TITLE-ABS (Consent AND Contraception) AND TITLE (law OR legal) AND PUBYEAR > 2000
TITLE-ABS (Consent) AND TITLE-ABS (Contraceptive AND Contraception) TITLE (law OR legal) AND PUBYEAR > 2000
TITLE-ABS("Emergency Contraception") and TITLE(law OR legal) AND PUBYEAR > 2000
TITLE-ABS ("Comprehensive Sexuality Education") AND TITLE-ABS (law OR legal) AND PUBYEAR > 2000
TITLE-ABS("Sexuality Education") and TITLE(law OR legal) AND PUBYEAR > 2000
TITLE-ABS ("HIV Testing") AND TITLE (law OR legal) AND PUBYEAR > 2000
TITLE-ABS ("HIV Counselling") AND TITLE (law OR legal) AND PUBYEAR > 2000
TITLE-ABS ("HIV Treatment") AND TITLE (law OR legal) AND PUBYEAR > 2000
TITLE-ABS ("HIV Care") AND TITLE (law OR legal) AND PUBYEAR > 2000
TITLE-ABS ("HIV" AND "Confidentiality") AND TITLE (law OR legal) AND PUBYEAR > 2000
TITLE-ABS ("HPV" AND "vaccine") TITLE (law OR legal) AND PUBYEAR > 2000

Supplemental File 2 - Semi-structured interview guide for key informant interviews

General Questions

1. How has the legal framework – laws relevant to Sexual and Reproductive Health (SRH) – been relevant to the work of the key informant?
2. How well do you think Colombia/Malawi/Uruguay/Zambia has managed to implement supportive laws around SRH that have a positive impact on SRH outcomes?
    a. What have been the facilitators in this process?
    b. What have been the challenges and how have these been overcome?
3. Are there any differences in implementation between different areas of SRH?
    a. What are the barriers and facilitators for implementation of laws relating to maternity care/contraception/abortion/sexuality education/HPV vaccine/HIV?
        i. Why do you think this are playing out this way?
        ii. What are the societal, social or structural facilitators or barriers?
4. Are there any contradictory laws affecting SRH? How do you work with that, or not?
5. Which population groups are still being left behind despite the existence of laws that are supportive of SRHR?
6. How do women in general around the country understand laws relating to SRH?
    a. Have you seen women's experience of SRH change at all when laws change?
7. Have you seen any changes in SRH indicators due to changes in law?

Different roles

1. What is the role of the government in implementing these laws?
    a. What have they done well and where is improvement needed?
2. What is the role of health worker unions or association in the implementation of SRH laws, if any?
3. What is civil society's role in implementation of these laws?
    a. Do civil society have space to do the work they want? Are they constrained at all by the government?
4. What role has the judiciary played in ensuring implementation of these laws?
5. How do political cycles affect all of this? Are there risks of regression?

Conclusion

1. If you could change or improve one thing about how SRH-related laws are implemented here, what would you change?
